# Benign Mixed Epithelial and Stromal Tumor of the Kidney

**DOI:** 10.1100/tsw.2006.115

**Published:** 2006-06-01

**Authors:** A. Işin Doğan Ekici, Sinan Ekici, Bora Gürel, Gülçin Altinok, Ilhan Erkan, Yücel Güngen

**Affiliations:** ^1^ Department of Pathology, Hacettepe University School of Medicine, Ankara, Turkey; ^2^ Department of Urology, Hacettepe University School of Medicine, Ankara, Turkey; ^3^ Department of Pathology, Yeditepe University School of Medicine, Istanbul, Turkey; ^4^ Department of Urology, Maltepe University School of Medicine, Istanbul, Turkey

**Keywords:** benign mixed epithelial and stromal tumor of the kidney, biphasic tumor, estrogen receptor, kidney, progesterone receptor

## Abstract

A 51-year-old, perimenopausal, female patient with 1-month history of right flank pain who was diagnosed with a renal mass and underwent nephron-sparing partial nephrectomy is presented. The renal mass was found to be a benign, biphasic tumor composed of an epithelial component, consisting of ducts of variable size scattered within a mesenchymal component, composed of spindle cells arranged in sheets and fascicles. No atypia, mitosis, or necrosis was found. The spindle component shows desmin, smooth muscle actin, and estrogen and progesterone receptor positivity immunohistochemically. The diagnosis of benign mixed epithelial and stromal tumor of the kidney is rendered. No recurrent disease has been detected during 2 years of follow up.

## INTRODUCTION

In 1998, a distinctive, benign, renal neoplasm composed of an epithelium with solid and cystic architecture within a spindle stroma was recognized by Michal and Syrucek. They named this tumor as "Benign Mixed Epithelial and Stromal Tumor" (BMEST)[1]. In 2000, Adsay et al. described the clinical and pathological features of 12 patients with BMEST. This rare, benign lesion of the kidney occurs almost exclusively in perimenopausal women. A strong association for long-term, oral estrogen therapy has also been postulated[2]. It is recognized that a number of these tumors have been reported in the literature under various names, including adult mesoblastic nephromas due to the predominating fascicular arrangement of smooth muscle cells, and some cases of BMEST that were primarily cystic have been diagnosed as cystic nephroma or cystic partially differentiated nephroblastoma[2].

## CASE REPORT

A 51-year-old, perimenopausal woman, receiving estrogen-containing hormonal replacement therapy for 2 years, with 1-month history of right flank pain was admitted to our institution. During the clinical evaluation, a right renal mass detected on ultrasonography was further described as a well-circumscribed, enhancing, inhomogeneous, solid, 3.6- × 2.6-cm measured mass. Laboratory tests including serum electrolytes, liver and renal function tests were normal. Urine analysis was unremarkable. She was also diagnosed as having a thalassemia trait during work-up. The patient underwent right partial nephrectomy for presumed renal cell carcinoma. During the operation, an intraoperative pathology consultation (frozen section) was performed. The frozen section revealed a benign tumor composed of epithelial and stromal elements. Surgical margins were free of neoplasm. For that reason, no further excision of the remaining kidney was required.

Macroscopically, the partial nephrectomy material measured 4.5 × 3.3 × 2.8 cm. A well-circumscribed, solitary, round, grey-tan colored mass, measuring 2.4 cm in diameter and small cystic spaces interspaced throughout the lesion could be discerned. The surgical margins were intact and a 0.2-cm-thick layer of renal cortex between the mass and the perirenal adipose tissue could be seen. Hematoxylin & Eosin stained sections from paraffin blocks including the whole neoplasm; renal cortex and surgical margins were prepared from the specimen for routine histological examination.

Histologically; the lesion was biphasic, consisting of an adenomatous component composed of tubular structures of varying diameter dispersed throughout a predominant stromal component formed by spindle-shaped cells arranged in fascicles (Fig. 1). No mitosis, necrosis, atypia, or extramedullary hematopoietic foci were seen. Surgical margins were lacking tumor histologically.

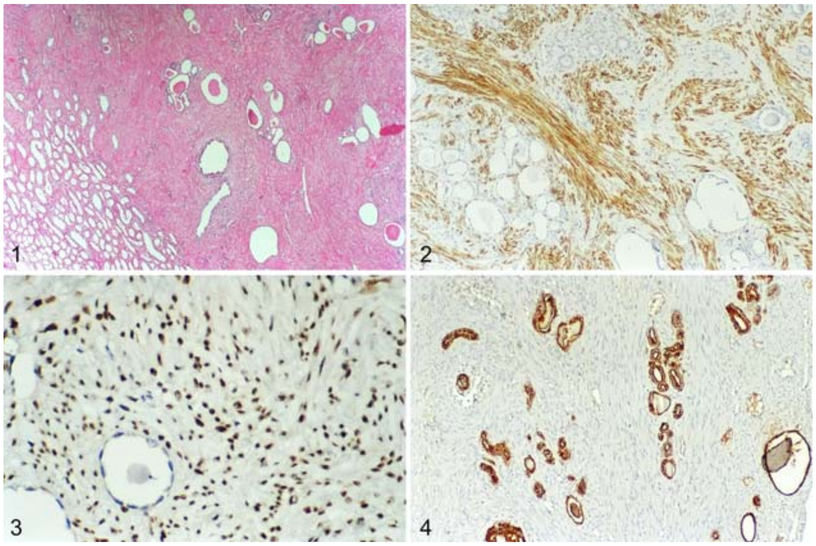

**FIGURE 1.** The biphasic lesion of kidney, with an adenomatous component composed of tubular structures of varying diameter dispersed throughout a predominant stromal component formed by spindle-shaped cells arranged in fascicles (×100, Haematoxylin & Eosin). **FIGURE 2.** Immunohistochemical expression of Smooth Muscle Actin in the stromal component (×150, SMA). **FIGURE 3.** Immunohistochemical nuclear positivity for Estrogen Receptor in the stromal cells (×200, ER). **FIGURE 4.** Epithelial component showing immunohistochemical expression of Epithelial Membrane Antigen (×100, CK).

Immunohistochemical studies revealed diffuse immunoreactivity for desmin (1/50, Neomarkers, CA, USA), smooth muscle actin (1/100, 1 A4, Neomarkers, CA, USA); estrogen receptor (ER, Neomarkers, CA, USA), and progesterone receptor (PR, Neomarkers, CA, USA) in the stromal component (Figs. 2 and 3). Epithelial component was immunoreactive for Cytokeratin (CK, AEl, AE3, Zymed, CA, USA) and Epithelial Membrane Antigen (EMA, Neomarkers, CA, USA) (Fig. 4). Both epithelial and stromal components were negative for HMB-45 (HMB45 + HMBSO, Neomarkers, CA, USA), CD34 Class-2 (CD34, DAKO, Denmark). The diagnosis of BMEST was rendered.

The patient was well and without evidence of disease during the follow-up visits after 2 postoperative years.

## DISCUSSION

BMEST is an individual, benign tumor of the kidney, composed of mesenchymal and stromal components. The mesenchymal component consists of spindle cells arranged in sheets and fascicles, showing variable degrees of fibroblastic, smooth-muscle, or myofibroblastic differentiation[1,2]. The epithelial component is variable, usually composed of dilated glands with round lumina, sometimes scattered throughout the stroma, but sometimes also seen in a closely clustered, back-to-back pattern. A few tumors with complex branching architecture and papillae have also been reported[2].

Because the lesion is very similar to the congenital form of mesoblastic nephroma, the first tumors reported were proposed to be "adult form of congenital mesoblastic nephroma". Although the morphology of spindle cells in BMEST and congenital mesoblastic nephroma are similar and both tumors show a biphasic pattern, the difference in age groups and gender predilection suggest that these two entities are, in fact, two different diseases[2,3]. Adsay et al. stated that the tumors in the literature that were reported as adult mesoblastic nephroma had cystic or other epithelial components. Therefore, those tumors should be regarded as examples of BMEST[2]. They proposed to discard the term "adult mesoblastic nephroma" since it has no relationship with congenital mesoblastic nephroma[2]. In addition, the recognition of metanephric stromal tumor and its distinction from mesoblastic nephroma made the age of the oldest patient with the diagnosis of mesoblastic nephroma, in the National Wilms' Tumour Study Pathology Center, 29 months[4]. This finding supports that mesoblastic nephroma is a neoplasm of infancy[4,5]. Finally, Pierson at al. showed that BMEST was genetically dissimilar to congenital mesoblastic nephroma[5].

In most of the reported cases, as in our case, the spindle-cell component of BMEST was strongly positive for both desmin and smooth muscle actin. Nuclear expressions of estrogen and progesterone receptors were also found in those spindle cells immunohistochemically[2].

BMEST occurs in adults and mostly in perimenopausal females similar to presented case[2,3,4,6]. Recently, a 12-year-old female patient with BMEST has been exceptionally reported[7].

Clinically, there is a significant female inclination: 11 of 12 cases in Adsay's study were women and the male patient was reported to be treated with diethylstilbestrol for 7 years following leuprolide acetate therapy for 4 years[2].

The strong predilection for middle-aged perimenopausal women and the association with prolonged oral contraceptive use have suggested a probable hormonal mechanism to be responsible for pathogenesis of BMEST[2,4,5,6,7,8].

Most patients present with flank pain, hematuria, or symptoms resembling genitourinary infections. Almost 20% of patients are asymptomatic and their tumors are detected incidentally.

Even though benign, a few cases of fatal malignant or aggressive mixed epithelial and stromal tumor of the kidney have also been reported[9,10]. Mixed epithelial and stromal tumor of the kidney is known to be positive for estrogen and/or progesterone receptors. However, immunohistochemical estrogen and progesterone receptor expression status have not been assured in those aggressive or malignant cases[9,10].

The pathogenesis of BMEST and its relationship to other renal neoplasms is still not well understood, but BMEST should be distinguished from other renal neoplasms. The differential diagnoses of mixed epithelial and stromal tumor of the kidney include rare biphasic renal tumors with malignant components such as sarcomatous transformation in cystic nephroma and cystic embryonal sarcoma. Those can be distinguished from BMEST by their malignant stromal components with atypia, mitosis, and necrosis[11].

Some benign tumors originated from the kidney such as metanephric stromal tumor, stromal rich angiomyolipoma, or solitary fibrous tumor may be excluded from BMEST by the help of immunohistochemistry. HMB-45 is often positive in angiomyolipoma. Solitary fibrous tumor and metanephric stromal tumor is sensitively positive for CD34 and negative for desmin[12]. BMEST is negative for HMB-45 and CD34, but stromal component of BMEST is positive for desmin.

Renal leiomyoma is another alternative benign tumor in the differential diagnosis of stromal-predominant BMEST[12]. BMEST differ from leiomyomas in that they have tubules within the stromal component.

In our opinion, the fact that the presented patient has a thalassemia trait was probably an incidental finding and it had no contributory effect on development of BMEST. Inconsistent with our opinion, none of the cases reported in the literature exhibited any hematological disorder.

In summary; we describe a case of "benign mixed epithelial and stromal tumor" preliminarily diagnosed during frozen section; the patient underwent minimal invasive nephron-sparing surgery and was free from disease during 2 years of follow-up. BMEST is an uncommon, benign tumor of the kidney that should be distinguished from other renal neoplasms. Intraoperative frozen section is helpful to rule out a malignancy and an effort for a nephron-sparing surgery is advisable. The prognosis is encouraging.
